# Network-guided neuromodulation for epilepsy: Unveiling the pathway to personalized therapy

**DOI:** 10.2478/jtim-2023-0101

**Published:** 2023-09-02

**Authors:** Peng Cao, Shun Gong, Liang Liu, Guobiao Liang

**Affiliations:** Department of Neurosurgery, General Hospital of the Northern Theater Command of Chinese People's Liberation Army, Shenyang 110000, Liaoning Province, China; Department of Neurology, General Hospital of the Northern Theater Command of Chinese People's Liberation Army, Shenyang 110000, Liaoning Province, China

## Introduction

Epilepsy, a chronic neurological disorder characterized by recurrent seizures, affects millions of individuals worldwide. Despite advancements in antiepileptic drugs and surgical interventions, a significant portion of patients continue to experience uncontrolled seizures, leading to a reduced quality of life. In recent years, the intersection of network neuroscience and neuromodulation has opened up promising avenues for personalized and targeted therapies for epilepsy. This editorial aims to synthesize and discuss the findings from multiple studies^[1, 2, 3, 4]^ that shed light on the role of specific brain regions in the epileptogenic network and their potential as neuromodulation targets. They have revealed that modulating specific brain regions within the epileptogenic network can yield therapeutic benefits for epilepsy patients. Multiple targets have been explored, including the anterior nucleus of the thalamus (ANT), the hippocampus, the subthalamic nucleus (STN), the cerebellum, and others. Each of these regions has unique connections with cortical and subcortical structures, making them potential nodes for seizure propagation.

## The anterior nucleus of the thalamus

The ANT has shown promise as a stimulation target, with studies demonstrating its involvement in seizure control. Preoperative network data have been used to predict the effectiveness of ANT deep brain stimulation (DBS) and responsive neurostimulation (RNS). Additionally, studies have highlighted the importance of individualized targeting within the ANT based on connectivity patterns and stimulation parameters. ANT, a component of the ‘extended hippocampal system’, inputs from the mamillary body, subicular and retrosplenial cortex and outputs to the medial prefrontal cortex. This network of ANT connects the hippocampus and thalamus through the mammillothalamic tract, fornix and cingulum. The ANT or this circuit neuromodulation may desynchronize, or even recalibrate, this network and interrupts the route of seizure propagation (Table 1). It is now a clinically approved and targeted site for open-loop stimulation and increasingly targeted for responsive neurostimulation. Some studies have shown that the anterior-ventral and anterior-medial ANT neuromodulation is more associated with responder status,^[5,6]^ as there are differences in connectivity of subnucleus-specific ANT, further studies are required to understand the network mechanisms may benefit from ANT neuromodulation.

**Table 1:** The role of specific brain regions in the epileptogenic network and their potential as neuromodulation targets

**Neuromodulation targets**	**Inputs**	**Outputs**	**Epileptogenic network**
Anterior Nucleus of the Thalamus	Mamillary body, subicular and retrosplenial cortex	Medial prefrontal cortex	Papez circuit
Hippocampus	Amygdala, piriform cortex, septal area	Mamillary body	Deep prepiriform, Septohippocampal
Subthalamic Nucleus	Corpus striatum	Thalamus, cingulate gyrus, cortex	Cortico- subcortical network
Cerebellum	Hippocampus, fairly global brain	Thalamus, superior colliculus, the pontine and medullary reticular formation, the locus coeruleus, and the amygdala	Locus coeruleus, the septum, and potentially the thalamus

## The hippocampus

The hippocampus, a critical structure in the brain’s memory and learning processes, has long been implicated in temporal lobe epilepsy (TLE). Researchers have observed alterations in the septo-hippocampal system and increased volumes of the septal nuclei in patients with TLE. The Hippocampus inputs from the amygdala, piriform cortex, septal area and outputs to the mamillary body. This network of hippocampus connects the medial temporal, inferior frontal lobes and medial frontal zone (Table 1). As the piriform cortex, it has been implicated as a key zone of seizure propagation, it has been demonstrated the hippocampus as an important node in the epileptogenic network was associated with a higher rate of seizure freedom,^[7,8]^ indicating the hippocampus is not only a seizure propagation zone, but also a site of epileptogenesis in TLE. What’s more, animal studies have shown that septal area stimulation can modulate hippocampal neuronal activity and terminate seizures, indicating its potential as a stimulation target. However, further research is needed to unravel the precise role of the hippocampus in the epileptogenic network and its therapeutic implications.

## The subthalamic nucleus

While the STN is primarily associated with Parkinson’s disease (PD), it has also been explored as a stimulation target in epilepsy.^[9]^ Studies have suggested a relationship between the STN and the motor cortex, with evidence of downstream propagation of epileptiform activity. STN inputs from the corpus striatum and outputs to thalamus, cingulate gyrus, cortex. This network of STN connects the substantia nigra, thalamus and the cortex. High-frequency stimulation of the STN has shown reductions in interictal spiking and high-frequency oscillations, indicating its involvement in seizure propagation.^[10]^ It inferred that STN acted on a ‘cortico-subcortical network’ by antidromic neuromodulation of the cortex. However, the exact mechanism of STN stimulation in epilepsy remains unknown and requires further investigation.

## The cerebellum and other targets

Early studies hinted at the potential of cerebellar stimulation in inhibiting seizures, but subsequent research has yielded mixed results. Traditionally, the cerebellum is not an area of the brain associated with epilepsy, Recent findings suggested that it can play an important role in epileptogenic network. The cerebellum inputs from the fairly global brain, hippocampus (epilepsy related), and outputs to the thalamus, superior colliculus, the pontine and medullary reticular formation, the locus coeruleus, and the amygdala. The changes in this network activity during seizures was associated with epilepsy. The cerebellum has not been acknowledged as a potential neuromodulation target for epilepsy until recently (Table 1). The use of closed-loop optogenetic, a more targeted approach, appeared to have greatly increased the efficacy of cerebellar neuromodulation in attenuating seizures. Recent researches renew this excite result in the possible cerebellar neuromodulation target for therapeutic intervention.^[11]^ Other potential targets, including the central lateral thalamus, pontine nucleus oralis, hypothalamus, and caudate nucleus, have also been explored to varying degrees. However, additional preclinical and clinical evidence is required to understand their role as seizure propagation points.

## Personalized, Network-Guided neuromodulation, mechanism, and ethics

Recent findings from multiple modalities of investigation, including scalp and invasive electroencephalography, diffusion and functional magnetic resonance imaging, and intracranial recordings from implanted devices, provided insights into the structural and functional networks associated with these propagation points. Epilepsy is typically the result of the networks that are recruited or traveled by the epileptiform discharges and the network hub analysis showed epileptogenic networks may be associated with multiple brain networks, including default mode network, frontoparietal control network, and cingulo-opercular network.^[12]^ The mechanism of the network hub alteration remains unknown and warrants further investigation. The epilepsy surgery is an attempt to sufficiently destroy the putative seizure-onset zone or structurally disconnect the bridging the epileptogenic and normal white matter. DBS has shifted to targeting the most influential downstream points in the epilepsy network, aiming to prevent further spread of epilepsy activity. RNS aims to inhibit the production of seizure by stimulating the “response” of seizure-onset zone to the epileptic activities recorded by seizure-onset zone.^[13]^ The ultimate goal is to employ preoperative network metrics to predict neuromodulation outcomes and personalize therapies. Studies have already demonstrated the ability to predict surgical outcomes based on preoperative network data. Retrospective studies have associated network connectivity measures with response to DBS and RNS, suggesting the potential for using preoperative network measures as biomarkers for patient selection and stimulation targeting. However, prospective studies and further validation are necessary to establish the utility of these approaches. With the progress of neuromodulation, it can also bring many unexpected consequences and even raise ethical issues. With the understanding of human brain function, it is possible to directly produce pleasure, anxiety, depression, and even violent aggression through stimulation. Such influence is related to the independence and dignity of individuals, and requires the government, academia, and industry to jointly consider the relevant ethical risks in order to truly promote the development and application of neuromodulation technology.^[14]^

## Conclusion

The convergence of network neuroscience and neuromodulation presents a promising direction for the treatment of epilepsy. By identifying specific brain regions within the epileptogenic network and understanding their connectivity patterns, personalized and targeted interventions can be developed. Future research should focus on refining stimulation parameters, investigating new targets, and harnessing machine learning algorithms to enhance our ability to predict stimulation outcomes. The advent of network-guided neuromodulation holds immense potential in transforming epilepsy therapy, offering new hope for patients living with uncontrolled seizures.
